# Diagnostic performance of various familial hypercholesterolaemia diagnostic criteria compared to Dutch lipid clinic criteria in an Asian population

**DOI:** 10.1186/s12872-017-0694-z

**Published:** 2017-10-16

**Authors:** Suraya Abdul-Razak, Radzi Rahmat, Alicezah Mohd Kasim, Thuhairah Abdul Rahman, Suhaila Muid, Nadzimah Mohd Nasir, Zubin Ibrahim, Sazzli Kasim, Zaliha Ismail, Rohana Abdul Ghani, Abdul Rais Sanusi, Azhari Rosman, Hapizah Nawawi

**Affiliations:** 1 Institute for Pathology, Laboratory and Forensic Medicine (I-PPerForM), University Teknologi MARA, 47000 Sungai Buloh, Selangor Malaysia; 2 Pathology Discipline, Faculty of Medicine, University Teknologi MARA, 47000 Sungai Buloh, Selangor Malaysia; 3 Primary Care Medicine Discipline, Faculty of Medicine, University Teknologi MARA, 68100 Selayang, Selangor Malaysia; 4 Cardiology Unit, Faculty of Medicine, University Teknologi MARA, 47000 Sungai Buloh, Selangor Malaysia; 5 Population Health and Preventive Medicine Discipline, Faculty of Medicine, University Teknologi MARA, 47000 Sungai Buloh, Selangor Malaysia; 6 Endocrinology Disciplines, Faculty of Medicine, Universiti Teknologi MARA (UiTM), 47000 Sungai Buloh, Selangor Malaysia; 7 National Heart Institute, No 145 Jalan Tun Razak, 50400 Kuala Lumpur, Malaysia

**Keywords:** Familial Hypercholesterolaemia, Simon Broome, Dutch Lipid Clinic Criteria, US Make Early Diagnosis to Prevent Early Deaths, Japanese FH Management Guideline Criteria

## Abstract

**Background:**

Familial hypercholesterolaemia (FH) is a genetic disorder with a high risk of developing premature coronary artery disease that should be diagnosed as early as possible. Several clinical diagnostic criteria for FH are available, with the Dutch Lipid Clinic Criteria (DLCC) being widely used. Information regarding diagnostic performances of the other criteria against the DLCC is scarce. We aimed to examine the diagnostic performance of the Simon-Broom (SB) Register criteria, the US Make Early Diagnosis to Prevent Early Deaths (US MEDPED) and the Japanese FH Management Criteria (JFHMC) compared to the DLCC.

**Methods:**

Seven hundered fifty five individuals from specialist clinics and community health screenings with LDL-c level ≥ 4.0 mmol/L were selected and diagnosed as FH using the DLCC, the SB Register criteria, the US MEDPED and the JFHMC. The sensitivity, specificity, efficiency, positive and negative predictive values of individuals screened with the SB register criteria, US MEDPED and JFHMC were assessed against the DLCC.

**Results:**

We found the SB register criteria identified more individuals with FH compared to the US MEDPED and the JFHMC (212 vs. 105 vs. 195; *p* < 0.001) when assessed against the DLCC. The SB Register criteria, the US MEDPED and the JFHMC had low sensitivity (51.1% vs. 25.3% vs. 47.0% respectively). The SB Register criteria showed better diagnostic performance than the other criteria with 98.8% specificity, 28.6% efficiency value, 98.1% and 62.3% for positive and negative predictive values respectively.

**Conclusion:**

The SB Register criteria appears to be more useful in identifying positive cases leading to genetic testing compared to the JFHMC and US MEDPED in this Asian population. However, further research looking into a suitable diagnosis criterion with high likelihood of positive genetic findings is required in the Asian population including in Malaysia.

## Background

Familial hypercholesterolaemia (FH) is an autosomal dominant disorder affecting between 1/200 to 1/500 individuals worldwide. The elevated serum cholesterol leads to more than 50% risk of fatal coronary artery disease in men and women [1]. FH occurs clinically in two forms namely, heterozygous FH and homozygous FH [2]. Patients with a mutation in both low-density lipoprotein receptor (LDLR) alleles are more severely affected than patients with a single mutant allele. Heterozygous FH has two to three fold elevations in plasma low-density lipoprotein (LDL-c) and typically begin to develop premature coronary artery disease (CAD) at the age of 30 to 40 years [3, 4]. In contrast, homozygous FH have six to ten times higher than the normal concentration of plasma LDL-c from birth and often have heart attacks during the first two decades of life [2, 5].

In the UK, the National Institute for Health and Care Excellence (NICE) recommends the Simon-Broome Register (SB) Register criteria [6] which includes raised cholesterol levels, family history of premature heart disease and hypercholestrolaemia, clinical characteristics, family history of premature CAD but also included a molecular diagnosis for confirmation of possible FH (Table 1). The Dutch Lipid Clinic Criteria (DLCC) is widely recommended for the rest of Europe. It is similar to the SB Register criteria but each criterion is given a weightage (Table 2) [7].

**Table 1:** Simon Broome Register Group of FH

**A definite diagnosis of FH requires:**
(a) Total cholesterol level above 7.5 mmol/L (290 mg/dl) in adults or a total cholesterol level above 6.7 mmol/L (260 mg/dl) for children under 16 OR LDL-c levels above 4.9 mmol/L (190 mg/dL) in adults (4.0 mmol/L in children) (either pretreatment or highest on treatment) PLUS
(b) tendon xanthomas in patient or relative (parent, child, sibling, grandparent, aunt, uncle) OR
(c) DNA-based evidence of an LDL receptor mutation or familial defective apo B-100
**Possible FH is defined as (a) above plus one (d) or (e):**
(d) Family history of myocardial infarction before age 50 in grandparent, aunt, uncle or before age 60 in parent, sibling or child.
(e) Family history of raised cholesterol in parent sibling or child, or level above 7.5 mmol/L (290 mg/dl) in grandparent, uncle, aunt

**Table 2:** Dutch Lipid Clinic network diagnosis of FH

Criteria	Points
Family History	
A First degree relative with known premature (<55 years men; < 60 years women) coronary disease and vascular disease OR LDL-c > 95^th^ percentile	1
B First degree relative with tendon xanthomata and/or arcuscornealis OR childhood (<18 years) with LDL-c > 95^th^ percentile	2
Clinical history	
A Patient with premature CAD (men <55, women <60 years)	2
B Patient with premature cerebral or PVD (men <55, women <60 years)	1
Physical Examination	
A Tendon xanthomas	6
B Premature arcus	4
Lab analysis A LDL – c > 8.5 mmol/L	8
B LDL-c 6.5–8.4 mmol/L	5
C LDL- c 5.0–6.4 mmol/L	3
D LDL – c 4.0–4.9 mmol/L	1
Functional	
DNA mutations	8
Definite FH: > 8 points, Probable FH: 6–8 points, Possible FH: 3–5	

Unlike the SB Register or the DLLC, the US Make Early Diagnosis to Prevent Early Deaths (US MEDPED) criteria defined FH based on age-specific and relative’s specific criteria for total cholesterol. The serum cholesterol cut-off points used in general populations are higher compared to serum cholesterol cut-off value when they had a first- and second- or third degree relatives with FH (Table 3).

**Table 3:** The US MEDPED Criteria

Age (year)	1st degree	2nd degree	3rd degree	General population
<20	5.7	5.9	6.2	7.0
20–29	6.2	6.5	6.7	7.5
30–39	7.0	7.2	7.5	8.8
≥40	7.5	7.8	8.0	9.3

The Japanese FH Management Criteria (JFHMC) recently defined FH into two groups of either homozygous FH or heterozygous FH. The homozygous FH must feature all of these criteria; serum total cholesterol of 15.5 mmol/L or more, presence of tendon xanthoma, premature CAD during childhood and a family history of heterozygous FH among the parents; while heterozygous FH is defined by LDL-c ≥ 4.7 mmol/L, presence of tendon xanthomas, and family history within the second-degree relatives with FH or premature CAD (Table 4).

**Table 4:** Diagnostic criteria for adult (15 years or older) homozygous FH by the Japanese FH Management Guideline Criteria

Hypercholesterolaemia (serum total cholesterol before treatment: ≥ 15.5 mmol/L)
Tendon xanthomata (tendon xanthoma on the dorsal hands, elbows, and knees, or Achilles tendon thickening^1^) or nodular xanthoma on the skin
Premature CAD during childhood
Parents’ family history of heterozygous FH.
**Diagnostic criteria for adult (15 years or older) heterozygous FH by the Japanese FH Management Guideline Criteria**
Hyper- LDL cholesteroalemia (LDL-C before treatment: ≥ 4.7 mmol/L) Tendon xanthomata (tendon xanthoma on the dorsal hands, elbows, and knees, or Achilles tendon thickening) or nodular xanthoma on the skin Family history within the second- degree relatives FH or premature CAD (men <55, women <60 years)

^1^Patients with Achilles tendon thickening (≥9 mm) on radiography should be regarded as having xanthoma

Despite established clinical assessment guidelines, most people with FH are undiagnosed or only diagnosed after their first coronary event [8]. Treatment with statin is effective in FH and early treatment delays or prevents the onset of CAD [9–11]. Hence, early identification of affected individuals is vital as treatment could substantially reduce the risk of coronary events. Most European countries diagnose less than 20% of all estimated cases [8], while in the UK, only 25% were diagnosed; with the remain undiagnosed until middle age [12]. In the USA, less than 10% of FH are formally diagnosed [13]. The readily available clinical guidelines for FH register have limited utility in case findings in their settings as both the DLCC and the SB Register criteria would require genetic testing. Genetic testing is recommended when a mutation is known within a family but only 10% to 40% of those with a clinical diagnosis of FH would have a mutation [8, 14]. Furthermore, genetic testing alone has become less favourable as a diagnostic criterion as the facility is not widely available and is expensive [14]. Therefore, case detections via personal and family history, physical examinations and other biochemical markers have been used to identify individuals in many countries including Malaysia. Currently, the prevalence of FH is still unclear in this country and there are no local criteria available to diagnose FH to be used by health care professionals. It is observed that the DLCC and the SB register criteria have been used invariably to diagnose FH in this country. However, diagnostic performance of all the criteria in detecting FH in Asian populations is unknown. This study aimed to examine the diagnostic performance of the SB Register criteria, the US MEDPED and the JFHMC compared to the DLCC. We also aimed to investigate the association between FH subgroup of the SB Register criteria, the US MEDPED and the JFHMC with the DLCC.

## Methods

### Study design

This is a cross sectional study involving 755 individuals with LDL-c level of ≥4.0 mmol/L recruited from multiple centres including the institution’s specialist primary care, lipids and cardiology clinics; National Heart Institute as well as community health screening programmes conducted from January 2011 to January 2015. Sample size was calculated using single proportion formula [15] based on proportion of positive FH detected using the SB Register criteria [14], US MEDPED [14] and JFHMC [16] considering 95% confidence interval, the required calculated sample size was 384. The exclusion criteria were subjects with secondary hypercholesterolaemia such as hypothyroidism, nephrotic syndrome, chronic kidney disease based on CKD EPI guidelines [17] and obstructive liver diseases. Eligible subjects were classified into “positive FH” subgroup and “negative FH” subgroup by the DLCC. Cases were identified as positive FH when the subjects achieved ≥3 points by the DLCC while subjects were classified as negative FH when the subjects achieved <3 points by the DLCC. Both groups were matched for age, gender, ethnicity, diabetes, hypertension and smoking status. Subsequently, all individuals were regrouped to be defined as “Yes FH” or “No FH” by the SB Register criteria, the US MEDPED and by the JFHMC. DLCC Positive FH subjects were then reclassified according to the subgroups of FH by the SB Register criteria, the US MEDPED and the JFHMC. For patients who were on lipid-lowering treatment at the time of measurement, pre-treatment LDL-c level was used, but if the pre-treatment level was not available, it was predicted using a correction table by Ellis et al. [18], which requires the information of current type of administered statin and its dose. Genetic mutation confirmation was not included in making the diagnosis of positive FH by the DLCC and SB Register criteria used. Family cascade screening was later performed for positive FH index cases identified by the DLCC criteria.

### Data collection

Subjects were given information on the study objectives and invited to participate. A standard questionnaire was used to obtain socio-demographic characteristics (age, gender, and ethnicity), lifestyle risk factors (smoking status), personal and family history of CAD. Anthropometric data including height and body weight to determine body mass index (BMI), waist circumference (WC) and blood pressure (BP) were also collected. The blood pressure was measured at least three times at 5 min apart on the right arm supported at heart level, using Omron automatic digital blood pressure monitor (Omron HEM-757). The average of the last 2 BP readings was used for analysis. Height and weight were measured by trained nursing staff using a balance beam scale with the subjects’ shoes removed. BMI was calculated as weight in kilograms divided by the square of height in meters (kg/m^2^). WC was measured to the nearest 0.5 cm using a measuring tape midway between the inferior margin of the last rib and the iliac crest in a horizontal plane. Fasting serum and plasma samples were collected for the measurement of lipid profile using automated analyzers (Roche, Cobas Integra 400, USA and Centaur XP, Siemens, USA).

### Statistical analysis

All statistical analysis was performed using IBM Statistical Program for Social Science Software (SPSS) ver. 16.0 for Windows. Variables with normal distribution were expressed as mean ± standard error of mean (SEM). The comparisons of FH detected by the different criteria used to diagnose FH were determined using chi-square test. *P*-values <0.05 were considered as significant. Sensitivity, specificity, positive, negative predictive and accuracy values were determined for positive FH diagnoses as defined by the positive FH by the DLCC against the SB Register criteria, the US MEDPED and the JHMC definition. Efficiency was defined as the sum of the correct diagnoses (positive and negative by the DLCC criteria) divided by all subjects. Significance level was set at *p*-value of <0.05.

## Results

A total of 415 subjects were classified as “positive FH” and 340 subjects as “negative FH” by the DLCC in the study. The baseline characteristics of the subjects in both groups were summarized in Table 5. It was found that the presence of CAD, xanthomas, corneal arcus, total cholesterol (TC) level and LDL-c level were significantly higher in positive FH group compared to negative FH group (*p* < 0.001).

**Table 5:** Baseline characteristics of subjects with DLCC positive and DLCC negative

Parameter	DLCC Positive *n* = 415	DLCC Negative *n* = 340	*P* value
^b^Age (Years)	47.5 ± 0.62	47.2 ± 0.7	Ns
^a^Gender	Male: 52.1	Male: 44.2	Ns
	Female: 47.9	Female: 55.8	
^a^Ethnicity	Malay: 75.2	Malay: 81.5	Ns
	Chinese: 16.1	Chinese: 10.9	
	Indian: 5.5	Indian: 3.2	
	Other: 3.2	Other: 4.4	
^a^Hypertension	19.6	15.7	Ns
^b^SBP (mmHg)	126.3 ± 1.2	125.4 ± 0.9	Ns
^b^DBP (mmHg)	74.9 ± 0.8	75.9 ± 0.7	Ns
^a^Diabetes	5.0	8.5	Ns
^b^Plasma glucose (mmol/L)	5.6 ± 0.2	5.7 ± 0.2	Ns
^a^Smoking	Yes: 14.2 No: 85.8	Yes: 17 No: 73	Ns
^b^BMI	26.5 ± 0.3	26.5 ± 0.3	Ns
^b^TC (mmol/L)	8.0 ± 0.07	6.5 ± 0.02	*
^b^TG (mmol/L)	2.0 ± 1.8	1.8 ± 1.0	Ns
^b^LDL (mmol/L)	5.8 ± 0.1	4.4 ± 0.1	*
^b^HDL (mmol/L)	1.3 ± 0.1	1.4 ± 0.1	Ns
^b^WC	88.6 ± 1.1	87.2 ± 0.6	Ns
^a^Xanthomas	29.4	0.0	*
^a^CA	20.2	0.5	*
^a^CAD	16.1	0.0	*

Table 6 showed the sensitivity and specificity of positive FH as defined by the SB Register criteria, the US MEDPED and the JFHMC against the DLCC as the reference criteria for diagnosis. The SB Register criteria was able to identify 51.1% positive FH out of the total DLCC defined cases. There was a high positive predictive value of 98.8% with a low efficiency value at 28.6%. The second best diagnostic performance was by the JFHMC with a sensitivity of 47.0%, a positive predictive value of 97.9% and efficiency value of 26.4%. The US MEDPED was only successful in diagnosing 25.3% of those diagnosed with FH by the DLCC. The negative predictive value was acceptable at 52.1%. The efficiency value was comparable with the value obtained with the SB Register criteria against the DLCC (26.4% vs. 28.6% respectively). It was also noted that from positive FH cases defined by the DLCC, the SB Register criteria identified the most number of positive FH compared to the US MEDPED or the JFHMC (212 vs. 105 vs. 195 respectively, *p* < 0.001). The SB criteria, US MEDPED and JFHMC identified similar number of negative FH cases in comparison to the DLCC (336 vs. 338 vs. 336 respectively, p < 0.001).

**Table 6:** Sensitivity, specificity, positive predictive value and negative predictive value for positive FH as defined by the SB, US MEDPED and JFHMC definitions against the DLCC definition

Definitions	DLCC								
	Yes FH	No FH	TOTAL	Sensitivity(%)	Specificity (%)	PPV (%)	NPV (%)	Efficiency (%)	p-value^†^
	Simon Broome								
Positive FH	212	4	216	51.1	98.8	98.1	62.3	28.6	<0.001*
Negative FH	203	336	539						
TOTAL	415	340	755						
	US MEDPED								
Positive FH	105	2	107	25.3	99.4	98.1	56.1	14.1	<0.001*
Negative FH	310	338	648						
TOTAL	415	340	755						
	JFHMC								
Positive FH	195	4	199	47.0	98.8	97.9	60.4	26.4	<0.001*
Negative FH	220	336	556						
TOTAL	415	340	755						

Significant = *p < 0.05; FH = Familial Hypercholesterolaemia; DLCC = Dutch Lipid Clinic Criteria; SB = Simon Broome’s Register Criteria; USMEDPED = US Make Early Diagnosis to Prevent Early Deaths criteria; JFHMC = Japanese Familial Hypercholesterolaemia Management Criteria; PPV: positive predictive value, NPV: Negative Predictive Value. ^†^ Pearson correlation

The percentage of subgroups for positive FH as defined by the SB Register criteria, the US MEDPED and the JFHMC against the subgroups as defined by the DLCC criteria is shown in Table 7. Among the definite FH classified by the DLCC criteria, there were 95.4% cases defined as definite FH by the SB Register criteria, 95.4% as heterozygous FH by the JFHMC and 50.8% as heterozygous FH by the US MEDPED criteria. Meanwhile among the unlikely FH as defined by the DLCC, there were 98.8% cases defined as “no FH” by the SB Register criteria, 99.4% as “no FH” by the US MEDPED and 98.8% as “no FH” by the JFHMC. Furthermore, among the possible FH by the DLCC, 79.2% were classified as “no FH” by the SB Register criteria, 92.5% as “no FH” by the US MEDPED and 84.6% as “no FH” by the JFHMC. Among the probable FH as defined by the DLCC, 53.3% were classified as definite FH by the SB Register criteria, 46.7% as heterozygous FH by the US MEDPED and 71.1% as heterozygous FH by the JFHMC.

**Table 7:** Percentage of subgroups for positive FH as defined by the SB, US MEDPED and JFHC against the subgroups for positive FH as defined by the DLCC definition

Definitions	DLCC				
	Definite FH *N* = 130 (%)	Probable FH *N* = 45 (%)	Possible FHN = 240 (%)	Unlikely FH N = 340 (%)	*p*-value^†^
Simon Broome					
Definite FH	124 (95.4)	24 (53.3)	0 (0)	0	<0.001*
Possible FH	2 (1.5)	12(26.7)	50 (20.8)	4 (1.2)	
No FH	4 (3.0)	9 (0.2)	190 (79.2)	336 (98.8)	
Total	130	45	240	340	755
US MEDPED					
Heterozygous	66 (50.8)	21 (46.7)	18 (7.5)	2 (0.6)	<0.001*
No FH	64 (49.2)	24 (53.3)	222 (92.5)	338 (99.4)	
Total	130	45	240	340	755
JFHC					
Heterozygous	124 (95.4)	32 (71.1)	37 (15.4)	4 (1.2)	<0.001*
Homozygous	2 (1.5)	0	0	0	
No FH	4 (3.1)	13 (28.9)	203 (84.6)	336 (98.8)	
Total	130	45	240	340	755

Significant = *p < 0.05; FH = Familial Hypercholesterolaemia; DLCC = Dutch Lipid Clinic Criteria; SB = Simon Broome’s Register Criteria; USMEDPED = US Make Early Diagnosis to Prevent Early Deaths criteria; JFHMC = Japanese Familial Hypercholesterolaemia Management Criteria. ^†^ Pearson correlation

Table 8 shows the association of classifications of subjects with no FH by the SB Register criteria, US MEDPED and JFHMC with subgroups as defined by the DLCC. Among the No FH by the SB Register criteria, 5.7% were definite FH, 30.9% were possible FH while 19.6% were probable FH by the DLCC. Among, the No FH by the US MEDPED, 91.4% were definite FH, 36.1% were possible FH and 52.2% were probable FH by the DLCC. Similarly, among the No FH by the JFHMC, 5.7% were definite FH, 33% were possible FH and 28.3% were probable FH by the DLCC. These associations between classifications of subjects with no FH by the SB Register, US MEDPED and JFHMC with subgroups as defined by the DLCC criteria were significant (*p* < 0.001).

**Table 8:** Association of No FH by the SB Register, USMEDPED and JFHMC with subgroups as defined by the DLCC

	DLCC				
	Definite FH n (%)	Probable FH n (%)	Possible FH n (%)	Unlikely FH n (%)	p-value^†^
No FH by SB Register	4 (5.7)	9 (19.6)	190 (30.9)	336 (33.3)	<0.001*
No FH by USMEDPED	64 (91.4)	24 (52.2)	222 (36.1)	338 (33.5)	
No FH by JFHMC	4 (5.7)	13 (28.3)	203 (33.0)	336 (33.3)	
Total	70	46	615	1010	

Significant = *p < 0.05; FH = Familial Hypercholesterolaemia; DLCC = Dutch Lipid Clinic Criteria; SB = Simon Broome’s Register Criteria; USMEDPED = US Make Early Diagnosis to Prevent Early Deaths criteria; JFHMC = Japanese Familial Hypercholesterolaemia Management Criteria. ^†^ Pearson correlation

## Discussion

At present, there have been various FH clinical criteria available for various populations but there is scarcity of known gold standard criteria to diagnose FH. To the best of our knowledge, this is the first study to demonstrate the ability of various clinical criteria to identify positive FH cases in an Asian population. We applied the DLCC as the reference diagnostic criteria to identify index cases because it is largely used in our clinical settings; its similarities to the SB Register criteria and its ability to predict presence of genetic mutations despite a weak phenotype-genotype concordance, [19]. Moreover, it uses lower cut of level for LDL-c with additional indicators such as arcus cornealis, peripheral vascular disease and premature cerebral disease when compared to other criteria, in which potentially greater FH cases will be identified.

In low-middle income Asian countries, routine genetic testing is technically demanding and costly. Similarly, genetic mutation confirmation parameter was not done for our study due to the financial constraint, which is commonly observed practice in Malaysia. Elucidating diagnostic performances of various clinical FH criteria, without the use of genetic testing is particularly useful for Malaysia and other Asian populations. Factors such as family or personal history of tendon xanthoma and LDL-c was found to be strongly associated with positive genetic diagnosis in a Spanish population, in which a thorough examination for tendon xanthoma in suspected FH patients were recommended prior genetic testing [14]. A recent study analyzing 5050 subjects from a UK database recommended an assessment tool algorithm in order to improve the detection of FH in primary care setting prior to referral for genetic testing [20]. The JFHMC is the only established FH diagnostic criteria for use in the Japanese population and genetic testing is not used to diagnose FH but without validation in other Asian population.

This study found that the SB Register criteria identified more individuals with FH compared to the US MEDPED and the JFHMC when assessed against the DLCC. All of the criteria had a high specificity but low sensitivity values. Nevertheless, the SB Register criteria had the highest sensitivity compared to the US MEDPED and the JFHMC (51.1% vs. 25.3% vs. 47.0%; *p* < 0.001). The low sensitivity or the poor performance in finding the true positive of FH cases according to the DLCC by the SB Register criteria could be explained by the difficulty of getting a complex family history in order to fulfill the diagnosis of positive FH. Similarly, the US MEDPED and the JFHMC require information on family history of premature CAD and relative’s serum cholesterol values to be classified as positive FH. Hence, complex clinical history may deter the performance of these criteria in detecting FH.

Our study found a high positive predictive (98.1% vs. 98.1% vs. 97.9%) but lower negative predictive values (62.3% vs. 56.1% vs. 60.4%) by the SB Register criteria, the US MEDPED and the JFHMC respectively. This could be explained by the fact that most of the subjects were identified from tertiary and lipid specialist care, in which prevalence of FH is higher than the general population. Therefore, the interpretation of the reliability of each criteria to predict true positive FH and true negative FH must be done with caution.

Although the US MEDPED is a simple diagnostic criterion, it was found to be successful in diagnosing only 25.3% of those diagnosed with FH when compared against the DLCC. It had the lowest sensitivity and efficiency value compared to other criteria. Furthermore, the majority of its No FH cases were reclassified as definite FH by the DLCC. The JFHMC had better sensitivity, specificity and efficiency value compared to the US MEDPED. The low diagnostic efficiency values in all of the criteria against the DLCC suggests that the SB Register, the US MEDPED and the JFHMC could not identify positive and negative FH accurately when compared to the DLCC. Future studies comparing these various criteria against results of genetic testing are required to strengthen the recommendation for genetic testing to confirm FH cases detected clinically.

To date, an excess of over 1000 genetic variants have been identified in the Low Density Lipoprotein-Receptor gene (LDLR), Apolipoprotein B-100 gene (APOB), or Proprotein Convertase Subtilisin/Kexin type 9 gene (PCSK9) [21]. Molecular diagnosis is often difficult except in populations where only a limited number of mutations predominate. We postulate that genetic testing would post a challenge in finding true FH cases due to the presence of vast spectrum of genetic variants in the multiethnic heterogenous population of Malaysia. Given that genetic testing is costly and not readily accessible in many countries, a need to identify the best phenotype indication to achieve accurate yield is required. This would be useful in determining the gold standard criteria for detecting FH in a Malaysian population.

## Conclusion

Our findings concluded that the SB Register criteria was better at detecting FH followed by the JFHMC and US MEDPED in this Asian population. The SB Register criteria has the highest sensitivity value with a comparably high specificity value when compared to the JFHMC and the US MEDPED. This implies SB Register criteria is more useful in identifying cases suitable for further genetic testing compared to the JFHMC and US MEDPED. Further research looking into a suitable diagnosis criterion with high likelihood of positive genetic findings is required in the Asian population including Malaysia.

## Abbreviations

BMI: Body mass index
BP: Blood pressure
CA: Corneal arcus
CAD: Coronary artery disease
DBP: Diastolic blood pressure
DLCC: Dutch lipid clinic criteria
DNA: Deoxyribonucleic acid
FH: Familial hypercholesterolaemia
HDL: High-density lipoprotein
JFHMC: Japanese Familial Hypercholesterolaemia Management criteria
LDL: Low-density lipoprotein
LDLR: Low-density lipoprotein receptor
NICE: National Institute for Health and Care Excellence
SB: Simon broome
SBP: Systolic blood pressure
SEM: Standard Error of Mean
SPSS: Statistical Program for Social Science Software
TC: Total cholesterol
TG: Triglyceride
US MEDPED: United State make early diagnosis to prevent early deaths
WC: Waist circumference
